# Sample size calculations for skewed distributions

**DOI:** 10.1186/s12874-015-0023-0

**Published:** 2015-04-02

**Authors:** Bonnie Cundill, Neal DE Alexander

**Affiliations:** MRC Tropical Epidemiology Group, Faculty of Epidemiology and Population Health, London School of Hygiene and Tropical Medicine, Keppel Street, London, WC1E 7HT UK

**Keywords:** Sample size, Generalized linear models, Power, Berry-Esséen theorem

## Abstract

**Background:**

Sample size calculations should correspond to the intended method of analysis. Nevertheless, for non-normal distributions, they are often done on the basis of normal approximations, even when the data are to be analysed using generalized linear models (GLMs).

**Methods:**

For the case of comparison of two means, we use GLM theory to derive sample size formulae, with particular cases being the negative binomial, Poisson, binomial, and gamma families. By simulation we estimate the performance of normal approximations, which, via the identity link, are special cases of our approach, and for common link functions such as the log. The negative binomial and gamma scenarios are motivated by examples in hookworm vaccine trials and insecticide-treated materials, respectively.

**Results:**

Calculations on the link function (log) scale work well for the negative binomial and gamma scenarios examined and are often superior to the normal approximations. However, they have little advantage for the Poisson and binomial distributions.

**Conclusions:**

The proposed method is suitable for sample size calculations for comparisons of means of highly skewed outcome variables.

**Electronic supplementary material:**

The online version of this article (doi:10.1186/s12874-015-0023-0) contains supplementary material, which is available to authorized users.

## Background

Sample size calculations estimate the required number of patients to meet a study’s objective(s). The method used to analyse the subsequent data will affect the actual power, although this dependence is often ignored in practice. Sample size calculations are often based on normal approximation, such as those described by Lachin [1], even for data which are not Gaussian and which are analysed using generalized linear models (GLMs) [2-6]. Some medical statistics textbooks which cover Poisson regression still obtain sample sizes for rates via a normal approximation [7-10]. Using a statistical method which does not correspond to that used for the sample size may result in the actual power differing from the nominal value.

Methods have been proposed for the specific cases of logistic [11-14] or Poisson [15] models, or both [16], or for the negative binomial [17], and for generalized linear models [18,19]. The more general methods concentrate on single or multiple continuous predictor variables and can be somewhat complex to use. In particular, not all of them yield an explicit formula for sample size. In the current paper we consider a comparison of two means, i.e. a dichotomous predictor variable. We obtain a general formula which encompasses, for example, the Poisson and binomial distributions, but concentrate on the negative binomial and gamma — partly replicating Zhu and Lakkis for the former [17] — because these can be used to model skewed data, for which normal approximations are less likely to be satisfactory. We apply these methods to examples based on actual studies, including the negative binomial distribution for hookworm egg counts, a potential vaccine trial endpoint, and the gamma distribution for concentrations of insecticide on bednets.

## Methods

We examine the magnitude of errors in normal approximations for discrete probability distributions. Then, using GLM theory, we then derive sample size formulae which are assessed using worked examples and simulations Additional file 1.

### Assessing the magnitude of error using normal approximations

The central limit theorem guarantees that, for a sufficiently large sample size, the sample mean has a distribution which is arbitrarily close to normal (Gaussian). To evaluate the adequacy of the normal approximation under specific circumstances, in terms of cumulative distribution functions, we used a) the Berry-Esséen theorem and b) computation of the specific distributions. All computing was done using R, version 2.15 or higher.

### Berry-Esséen theorem

Let *R*_1_*,R*_2_*,....,R*_*n*_ be independent and identically distributed (iid) zero-mean random variables with positive variance σ^2^. Defining ${S}_n={\displaystyle {\sum}_{k=1}^n{R}_k/\sigma \sqrt{n}}$ as the standardised mean of the random variables, *F*_*n*_(*y*) as the cumulative distribution function (CDF) of *S*_*n*,_ and Φ as the CDF of the standard normal distribution, the Berry-Esséen theorem [20] states

1 $$ \left|{F}_n(y)-\Phi (y)\right|\le \frac{C\rho }{\sigma^3\sqrt{n}} $$

where *C* is a distribution-independent positive constant, and *ρ* < ∞ is the absolute third central moment, Ε(|*R* − Ε(*R*)|^3^), which equals Ε(|*R*|^3^) thanks to the specification of zero mean. Values of *C* have decreased markedly from Esséen’s original bound of 7.59 [20] to 0.4690 obtained by Shevtsova in 2013 [21]. For Poisson sums, including the Poisson itself, and the negative binomial as a mixture of Poissons, this can be replaced by 0.3051 [22]. More precise values are also available for the special cases of the binomial distributions with parameter 0.5 [23] or with denominator 1 [24], although the latter is applicable only to sample sizes of at least 200.

The Berry-Esséen approach can be used even when direct calculation from the distribution is not feasible. The bound can be expressed in terms of the third non-absolute central moment and a finite sum (see Additional file 2). Such bounds are one way to assess the adequacy of the normal distribution assumptions implicit in common sample size methods. In the following section we describe a potentially more robust sample size approach.

### Sample sizes from generalized linear model theory

Generalized linear models are for vectors of independent responses, *Y*_*i*_(*i =* 1*,…,N*), arising from an exponential family distribution. Such distributions include the Poisson, binomial and gamma, as well as the negative binomial if its *k* parameter is assumed fixed [25,26]. Covariates *x*_*ij*_ enter the model as linear combinations with unknown regression coefficients *β*_*j*_ and can be written as

$$ {\eta}_i={\displaystyle \sum_{j=1}^p}{\beta}_j{x}_{ij} $$

where η_i_ is related to *μ*_*i*_, the mean of *Y*_*i*_, via the link function *g:η*_*i*_*= g(μ*_*i*_*)*.

The sample size for a hypothesis related to the mean of such a distribution can be calculated from the variance of its maximum likelihood estimate (MLE), on the scale of the link function. The covariance matrix of the parameter estimates for GLMs is approximately

2 $$ {\left({X}^TWX\right)}^{-1} $$

where *X* is the design matrix and *W* is the diagonal matrix of weights [27]. We need to know how the sample size affects the variance of the parameter estimate. When comparing the means of two groups of size *N*_0_ and *N*_1_ (with *N*_0_ + *N*_1_ = *N*), *X* has two columns and *N* rows. The first column, corresponding to the intercept, is all 1′s, and the second column is *N*_0_ zeros and *N*_1_ 1′s. *W* is defined by

3 $$ W=\frac{{\left(\frac{d\mu }{d\eta}\right)}^2}{V\left(\mu \right)} $$

where *V* (*μ*) is the variance function relating the mean and variance of *Y* [27]. The diagonal of *W* is composed of *N*_0_ copies of *w*_0_ and *N*_1_ copies of *w*_1_, in an obvious notation. To compare the two means, we are interested in the second diagonal element of the 2 × 2 matrix given by equation (2). Some basic matrix algebra shows that this element is (*N*_0_*w*_0_)^−1^ + (*N*_1_*w*_1_)^−1^.

For the sample size of this comparison, we apply principles outlined by Lachin [1]. His notation uses subscripts 0 and 1 for the null and alternative hypotheses, which here we will change to *O* and *A*, using 0 and 1 instead to refer to the two groups being compared: 0 for reference or control, and 1 for intervention. We will also use *λ* rather than *μ* as a generic parameter, using the latter to denote the mean. We will also use a different subscript notation for standard normal deviates, so that *z*_*p*_ means the standard normal deviate for lower tail area *p*. Our statistic (*X* in Lachin’s notation) is the estimate of the difference in transformed means obtained by GLM. The transformation is typically log, or logit for binomial. The mean of this statistic is *λ*_*O*_ under the null hypothesis and *λ*_*A*_ under the alternative hypothesis, with the standard deviation being *Σ*_*O*_ and *Σ*_*A*_. Lachin’s equation 1 then becomes

4 $$ \left|{\lambda}_A-{\lambda}_O\right|={z}_{1-\frac{\alpha }{2}}{\Sigma}_O-{z}_{1-\beta }{\Sigma}_A $$

Following Lachin again, we will denote the proportions in the groups by *Q*_0_ = *N*_0_/*N* and *Q*_1_ = =*N*_1_/*N*. Our approach is to apply a normal approximation on the scale of the link function. This is often the log, although, with the identity link, more familiar equations are obtained. We consider two approaches for estimating the variance under the null hypothesis. One is to use the reference value in both groups: following Zhu and Lakkis [17], we call this method 1. Using the above matrix algebra, *Σ*_*Ο*_ equals

$$ \begin{array}{l}\sqrt{\frac{1}{Q_1N}\frac{V\left({\mu}_0\right)}{{\left(d\mu /d\eta \Big|{}_{\mu ={\mu}_0}\right)}^2}+\frac{1}{Q_0N}\frac{V\left({\mu}_0\right)}{{\left(d\mu /d\eta \Big|{}_{\mu ={\mu}_0}\right)}^2}}\\ {}=\sqrt{\frac{1}{N}\frac{V\left({\mu}_0\right)}{{\left(d\mu /d\eta \Big|{}_{\mu ={\mu}_0}\right)}^2}\left(\frac{1}{Q_1}+\frac{1}{Q_0}\right)}\end{array} $$

and *Σ*_*Α*_ equals

$$ \sqrt{\frac{1}{Q_1N}\frac{V\left({\mu}_1\right)}{{\left(d\mu /d\eta \Big|{}_{\mu ={\mu}_1}\right)}^2}+\frac{1}{Q_0N}\frac{V\left({\mu}_0\right)}{{\left(d\mu /d\eta \Big|{}_{\mu ={\mu}_0}\right)}^2}} $$

Hence, for method 1, we obtain

5 $$ \sqrt{N}=\frac{Z_{1-\frac{\alpha }{2}}\sqrt{\left(\frac{1}{Q_1}+\frac{1}{Q_0}\right)\frac{V\left({\mu}_0\right)}{{\left(d\mu /d\eta \Big|{}_{\mu ={\mu}_0}\right)}^2}}+{Z}_{1-\beta}\sqrt{\frac{1}{Q_1}\frac{V\left({\mu}_1\right)}{{\left(d\mu /d\eta \Big|{}_{\mu ={\mu}_1}\right)}^2}+\frac{1}{Q_0}\frac{V\left({\mu}_0\right)}{{\left(d\mu /d\eta \Big|{}_{\mu ={\mu}_0}\right)}^2}}}{g\left({\mu}_0\right)-g\left({\mu}_1\right)} $$

Zhu and Lakkis [17] find that the test characteristics are generally better if, instead, *μ*_1_ is used for the intervention arm under the null hypothesis (‘method 2’), so *Σ*_*Ο*_ equal *Σ*_*Α*_, and

6 $$ \sqrt{N}=\frac{\left({Z}_{1-\frac{\alpha }{2}}+{Z}_{1-\beta}\right)\sqrt{\frac{1}{Q_1}\frac{V\left({\mu}_1\right)}{{\left(d\mu /d\eta \Big|{}_{\mu ={\mu}_1}\right)}^2}+\frac{1}{Q_0}\frac{V\left({\mu}_0\right)}{{\left(d\mu /d\eta \Big|{}_{\mu ={\mu}_0}\right)}^2}}}{g\left({\mu}_0\right)-g\left({\mu}_1\right)} $$

Equations (5) and (6) are general, with special distributional cases being easily determined. We will use equation (6) except when referring to previous work based on method 1.

### Negative binomial distribution

The negative binomial distribution is a generalization of the Poisson for count data, with an additional parameter (*k*) which can describe over-dispersion [28]. Small *k* implies a large variance and as *k* → ∞ the distribution tends to Poisson. We derive results first for the negative binomial distribution, then for the Poisson as a limiting case. Let *Y* be a random variable which follows the negative binomial distribution with population mean *μ* and dispersion parameter *k*, with the variance function being V(*μ*) = *μ* + (*μ*^2^/*k*) and density as shown in Additional file 3. Analysis by GLM usually employs a natural logarithm link function [25] for which *dμ/dη = μ*. Substituting into equation (5) gives

7 $$ \sqrt{N}=\frac{Z_{1-\frac{\alpha }{2}}\sqrt{\left(\frac{1}{\mu_0}+\frac{1}{k_0}\right)\left(\frac{1}{Q_1}+\frac{1}{Q_0}\right)}+{Z}_{1-\beta}\sqrt{\frac{1}{Q_1}\left(\frac{1}{\mu_1}+\frac{1}{k_1}\right)+\frac{1}{Q_0}\left(\frac{1}{\mu_0}+\frac{1}{k_0}\right)}}{ \log \left({\mu}_0\right)- \log \left({\mu}_1\right)} $$

For the special case of equal sample sizes and (*Q*_0_ = *Q*_1_ = 0.5) and *k* parameters (*k*_0_ = *k*_1_) this reduces to the equation by Brooker et al. [29]. Using equation (6) instead gives:

8 $$ \sqrt{N}=\frac{\left({Z}_{1-\frac{\alpha }{2}}+{Z}_{1-\beta}\right)\sqrt{\frac{1}{Q_1}\left(\frac{1}{\mu_1}+\frac{1}{k_1}\right)+\frac{1}{Q_0}\left(\frac{1}{\mu_0}+\frac{1}{k_0}\right)}}{ \log \left({\mu}_0\right)- \log \left({\mu}_1\right)} $$

A normal approximation can be obtained by applying equation (6) on the identity scale, with variances equal to ${\mu}_i+{\mu}_i^2/{k}_i\left(i=0,1\right)$:

9 $$ \sqrt{N}=\frac{\left({Z}_{1-\frac{\alpha }{2}}+{Z}_{1-\beta}\right)\sqrt{\frac{1}{Q_1}\left({\mu}_1+\frac{\mu_1^2}{k_1}\right)+\frac{1}{Q_0}\left({\mu}_0+\frac{\mu_0^2}{k_0}\right)}}{\mu_0-{\mu}_1} $$

We used simulation to estimate the actual power sample sizes obtained from equations (8) and (9), by generating repeated datasets of the calculated sizes and analysing them by GLM and Wald tests. We also used likelihood ratio tests, with similar results, unless where commented. For this we used the rnegbin and glm.nb function of the MASS package in R.

### Poisson distribution

Let *Y* be a random variable denoting the number of events per unit time (for example, per study duration) then *Y* follows the Poisson distribution with mean *μ*. By letting *k* tend to infinity in equation (8), or, equivalently, from equation (6) with log link and *V*(*μ*) = *μ*, we obtain:

10 $$ \sqrt{N}=\frac{\left({Z}_{1-\frac{\alpha }{2}}+{Z}_{1-\beta}\right)\sqrt{\frac{1}{Q_1}\left(\frac{1}{\mu_1}+\frac{1}{k_1}\right)+\frac{1}{Q_0}\left(\frac{1}{\mu_0}+\frac{1}{k_0}\right)}}{ \log \left({\mu}_0\right)- \log \left({\mu}_1\right)} $$

This is compared by simulation, for the case *Q*_0_ = *Q*_1_ = 0.5 (equal size arms), with the following normal approximation, on the scale of the identity link, obtained from equation (9) by again letting *k* tend to infinity:

11 $$ \sqrt{N}=\frac{\left({Z}_{1-\frac{\alpha }{2}}+{Z}_{1-\beta}\right)\sqrt{2\left({\mu}_1+{\mu}_0\right)}}{\mu_0-{\mu}_1} $$

This is also used, for example, by Kirkwood & Sterne [7], except that here we include a factor of 2 inside the square root to obtain the total study size.

### Binomial distribution

Let *Y* be a binomial random variable denoting the number of successes in *d* independent Bernoulli events, each with probability *μ*. The most common situation is to have *d* = 1, with each unit (person) having a response of 1 or 0 (e.g. positive or negative). An assumption of *d* = 1 may explain why the literature does not always show *d* in the variance function: we follow Fox [30] in using *V*(*μ*) = *μ*(1-*μ*)/*d*. For the canonical logit link, *dμ*/*dη* = *μ*(1-*μ*), so, from equation (6), we obtain

12 $$ \sqrt{N}=\frac{\left({Z}_{1-\frac{\alpha }{2}}+{Z}_{1-\beta}\right)\sqrt{\frac{1}{Q_1}\frac{1}{\mu_1\left(1-{\mu}_1\right)}+\frac{1}{Q_0}\frac{1}{\mu_0\left(1-{\mu}_0\right)}}}{\sqrt{d}\left(\mathrm{logit}\left({\mu}_0\right)-\mathrm{logit}\left({\mu}_1\right)\right)} $$

On the scale of difference in proportions (identity link), the corresponding equation is:

13 $$ \sqrt{N}=\frac{\left({Z}_{1-\frac{\alpha }{2}}+{Z}_{1-\beta}\right)\sqrt{\mu_1\left(1-{\mu}_1\right)\frac{1}{Q_1}+{\mu}_0\left(1-{\mu}_0\right)\frac{1}{Q_0}}}{\sqrt{d}\left({\mu}_0-{\mu}_1\right)} $$

This differs from the Lachin’s equation (12), and that of Kirwood and Sterne, both of which have *Z*_*α*_ multiplied by a function of $\overline{\pi}\left(1-\overline{\pi}\right)$, where $\overline{\pi}$ is an average of the *μ*_0_ and *μ*_1_. Some outcomes, in particular the occurrence of a given condition, could be quantified either as a Poisson rate (events per unit time, with rate *μ*) or as a binomial proportion (fraction of people experiencing the condition in a given period *T*). These options can be linked mathematically, with the latter probability equalling 1-*e*^-*μT*^. This relation can, in turn, be used to compare the power or sample size for quantifying a given scenario as either a rate or proportion. In this case the rate is the more powerful option [31]. This is to be expected, since the proportion loses information by considering all those with one or more events as a single category.

### Gamma distribution

The gamma is a two-parameter continuous distribution family over positive values. Special cases include the exponential distribution, and the sum of identical independent exponentials. In applications it typically models right-skewed data [32]. If *Y* is such a random variable with shape parameter *κ* and scale parameter *θ,* then *E*(*Y*) = *κθ* ≡ *μ* and *V*(*μ*) = *κθ*^2^ = *μ*^2^/*κ* [33]. Here we use the logarithmic link, although the reciprocal is canonical. Hence *dμ*/*dη =* and *w*_*i*_ = *μ*^2^/(*μ*^2^/*κ*_*i*_) = *κ*_*i*_ so equation (6) becomes

14 $$ \sqrt{N}=\frac{\left({Z}_{1-\frac{\alpha }{2}}+{Z}_{1-\beta}\right)\sqrt{\frac{1}{Q_1{\kappa}_1}+\frac{1}{Q_0{\kappa}_0}}}{ \log \left({\mu}_0\right)- \log \left({\mu}_1\right)} $$

## Results

### Berry-Esséen bounds

**Figure 1:**
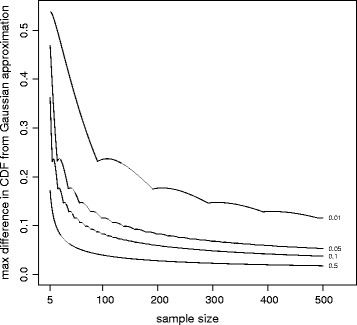
**Maximum difference between the approximating normal CDF and the computed binomial CDF, for varying sample sizes and sample proportion,** ***μ*** **.**

For the example of a fixed sample size of 100, the Berry-Esséen bounds are shown in the Table 1, along with corresponding values based on computation of the non-Gaussian CDFs. As expected, both methods show the normal approximation to be better for larger means. The Berry-Esséen bounds are often much wider than those obtained from explicit computation. Hence we concentrate on the latter approach. Figure 1 shows the results for binomial distributions of varying sample size and proportion (*μ*). As expected, the discrepancy in the CDF of the normal approximation is generally larger for smaller sample sizes and values of *μ* further from 0.5. The differences are non-negligible for parameter values found in some research studies, in particular for small values of *μ*, say between 1 and 5%, which would be expected to approximate Poisson. This tends to sustain a concern that power calculations based on normal approximations may not be accurate.

**Table 1:** **Maximum discrepancy in the approximating normal CDF, for sample size 100, in terms of Berry-Esséen bounds, and via computation**

**Distribution**	**Parameter estimates**		**Maximum error**	
			**Berry-Esséen**	**Exact CDF**
**Negative Binomial**	***k***	***μ***		
	0.05	0.05	28.9%	12.5%
		0.1	27.9%	9.8%
		10	27.3%	6.1%
		50	27.3%	6.0%
	0.1	0.05	22.3%	11.9%
		0.1	20.6%	8.8%
		10	19.5%	4.4%
		50	19.5%	4.4%
	0.5	0.05	15.7%	11.3%
		0.1	12.5%	8.3%
		10	9.4%	2.1%
		50	9.4%	1.9%
**Poisson**		***μ***		
		0.05	13.7%	11.6%
		0.1	9.8%	8.2%
		10	4.9%	3.2%
		50	4.9%	1.5%
**Binomial**		***μ***		
**(** ***d*** **= 1)**		0.05	19.5%	11.6%
		0.1	12.8%	8.3%
		0.5	4.7%	4.0%

### GLM approach for negative binomial distribution

We first revisit the example of Brooker et al. [29], which was motivated by the Human Hookworm Vaccine Initiative (HHVI). The degree of hookworm morbidity depends on the numbers of parasites in the intestines. Hence a quantitative endpoint is of interest for vaccine trials, and one option is the faecal egg count per Kato Katz slide. The negative binomial is often a good approximation to the distribution of such data, and the mean is a suitable summary measure [34]. For this, *μ*_1_ = 50, *μ*_0_ = 71.4 (30% vaccine efficacy), *k*_0_ = *k*_1_ = 0.33, *Q*_0_ = *Q*_1_ = 0.5, a null hypothesis of both means being equal to 71.4, 90% power and 5% significance level (two-sided). From equation (7) we again obtain a sample size of 505 per arm. From equation (8) we obtain 505 once more. This is because the methods differ in terms of the form 1/*μ* + 1/*k* and, for this example, 1/*k* dominates 1/*μ*, and *k* did not change. With the same parameter values, the normal approximation in equation (9) gives 531 per arm.

**Figure 2:**
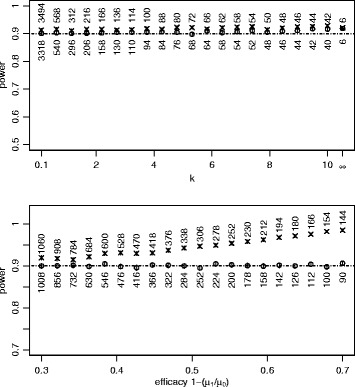
**Negative binomial sample size.** Each vertical axis shows power estimated by simulation for comparing two means and 90% nominal power. Each data point is based on 10,000 simulated datasets with two arms of equal size (*Q*_0_ = *Q*_1_ = 0.5), the ° symbol shows results for logarithmic link, equation (7), and × for identity link, equation (9). The vertical lines show the 95% confidence interval for the proportion of datasets reaching 5% two-sided significance level when analysed by GLM with logarithmic link, i.e. a measure of Monte Carlo error. The number beside each plot symbol is the sample size calculated by the relevant method. In the upper panel, *μ*_0_ = 71.4, *μ*_1_ = 50 (30% vaccine efficacy ) and various values of *k* (assumed equal in both arms) on the horizontal axis. In the lower panel, *μ*_0_ is as before, *k* is 0.33 and various values of efficacy are used (horizontal axis) with *μ*_1_ = *μ*_0_ × (1-efficacy).

Two sets of simulations were done: a) *k* was allowed to vary from 0.1 to 10, with the Poisson as a final limiting case (*k =* ∞); b) the efficacy, i.e. 1-(*μ*_1_/*μ*_0_), was allowed to vary from 0.3 to 0.7. Otherwise the parameters were held constant. The results are shown in Figure 2, where each data point is based on 10,000 simulations. For 30% vaccine efficacy, using the log link maintains close to the nominal power and the identity link is only slightly conservative (upper panel). As the efficacy, and the difference between the means, increases, the log link still maintains close to the nominal power whereas the identity link over-estimates the sample size, by more than 50% for the largest values of efficacy (lower panel).

### GLM approach for Poisson distribution

**Figure 3:**
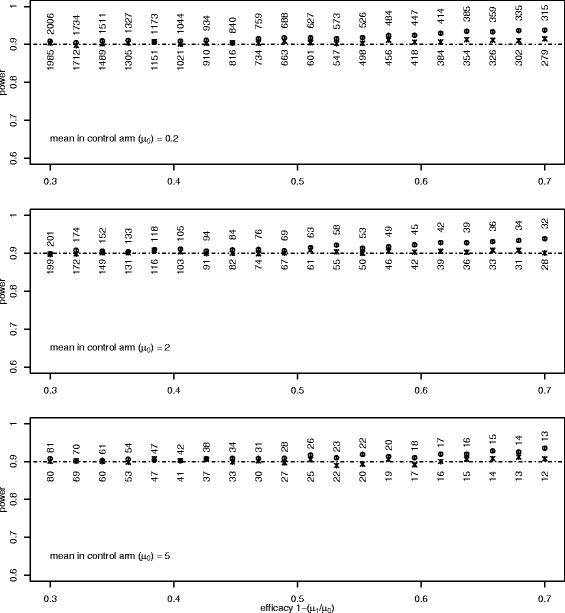
**Poisson.** This is similar to the lower panel of Figure 2, with each panel comparing two Poisson means. In each panel, the value of *μ*_0_ is shown, and *μ*_1_ = *μ*_0_ × (1-efficacy).

Equations (10), on the log scale, and (11), on the untransformed scale, were compared, with the power again set at 90%, and with three values of the mean in the control arm (*μ*_0_): 5, 2 and 0.2. Again using 10,000 simulations for each combination, the results are shown in Figure 3. The two methods are similar, and both slightly conservative for higher efficacies; the log link slightly more so.

### GLM approach for binomial distribution

Similar simulations were done for equations (12) and (13) with *d* = 1 and various values of *μ*_0_ and efficacy (1 minus the odds ratio). As before, data for each set of values was simulated 10,000 times. For *μ*_0_ = 0.5 both methods give close to nominal power. For *μ*_0_ = 0.1 and 0.05, the pattern is similar to the smaller Poisson means, with both being slightly conservative for higher efficacies; the logit link slightly more so (not shown). The simple dependency of the equations on *d* means that similar patterns were seen for *d* equal to 5 and 10 (not shown).

### GLM approach for gamma distribution

**Figure 4:**
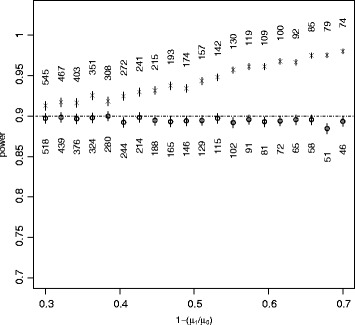
**Gamma.** Similar to the previous two figures but comparing means of two gamma distributions, with parameters based on a study of the insecticide deltamethrin on hammock nets.

Here we use an example based on concentrations of the insecticide deltamethrin on hammock nets in the Colombian Amazon [35], the mean being 8.46 mg/m^2^, and *κ* estimated as 0.639. As before, we compare the power of sample sizes from equation (14) with those from the corresponding normal approximation on the original scale. The results are shown in Figure 4. As in Figure 2, the sample size calculated on the scale of the link function maintains close to nominal power, while the normal approximation over-estimates the necessary sample size, by 50% or more for the larger differences in means.

In this case, the likelihood ratio test resulted in higher estimated powers for both tests (not shown). Since the sample size inputs were the same for both test methods, the difference scale again had appreciably more power than the logarithmic scale.

### Summary of simulation results

For the Poisson and binomial distributions, the results show little or no advantage for sample size calculations on the scale of the link function, i.e. log rates or log-odds, as opposed to the difference in rates or in proportions. By contrast, for the negative binomial and gamma distributions, which have additional parameters which can reflect skewness, sample size calculations based on differences in means can be very conservative, giving larger numbers which substantially exceed the required power. Sample size calculations on the log scale, however, retain close to the nominal power for the examples studies.

## Discussion

Normal approximations to distributions are often used to estimate sample sizes for discrete data, even when the data are to be analysed by generalized linear models. As well as being logically inconsistent, the magnitude of error is potentially large, judging by the discrepancies in CDF between the normal approximation and the exact distributions, whether assessed by the Berry-Esséen theorem or directly from distribution functions. This tends to sustain concerns about lack of robustness of normal approximations. Berry-Esséen and related theorems can, in principle, be used to estimate the speed of convergence of the normal approximation to that specified by the central limit theorem [20,22]. However, their bounds proved to be often markedly wider than those obtained from computing the CDF of the relevant distribution.

Considering robustness at the analysis stage, the *t* test performs well under certain large departures from normality [36]. Nevertheless, it is liable to break down when ‘skew is severe or when population variances and sample sizes both differ’ [37,38]. These are the circumstances for which we suggest the methods presented in the current paper are most suitable. The negative binomial and gamma distributions can capture severe skewness, and their variances differ between samples if the means do, due to their variance functions (*V*(*μ*)). We have used examples related to parasitology and entomology, but numbers of events, such as clinic visits or epileptic fits can also yield skewed count data. On the other hand, if a particular distribution family cannot be assumed then methods are available for sample sizes for non-parametric tests [39].

Under the simulation scenarios examined, where the proposed and standard methods differ, the latter tend to be conservative. The fact that many trials do not recruit their target sample sizes [40] may suggest acquiescence in such sample size over-estimation. However, compliance with the ethical requirement to avoid unnecessary exposure to novel treatments [41] — both to reduce potential harms, and to speed the acceptance of favourable interventions — would seem to be better assured by improving both the mathematical estimation and the recruitment process, rather than anticipating a tendency for their errors to cancel.

Some previous sample size methods for GLMs concentrate on single or multiple continuous predictor variables. They tend to be complex and do not always involve an explicit expression for the sample size. Here we have obtained simple equations for the comparison of two means, which is the most common situation for clinical trials. For the negative binomial, the method shown here corresponds to Zhu and Lakkis ‘Approach 2’ [17], although we allow *k* to differ between the arms (our *k* is the reciprocal of Zhu and Lakkis’). The approach was motivated by the need to plan later phase trials of vaccines against hookworm [29], a disease whose morbidity is related to infection intensity which in turn is measured by faecal egg counts. The high skewness of these counts seemed to preclude the use of normal approximations [34]. Negative binomial modelling may be appropriate for other parasite species [42] and other types of count [28], including insects [43] disease episodes [44], lesions [45], and cells [46]. For this distribution, there is a visible correspondence between the current formulae and that given by Krebs for estimating a mean with given percentage precision [47]. In fact our approach does not require specification of the complete distribution but only the link and variance functions. For the gamma, another example in the hookworm vaccine trials was the use of faecal heme as a candidate secondary endpoint. This is likely to be roughly proportional to the number of adult worms in the gut, and a gamma distribution was found to be a good fit to available data. More generally, gamma GLMs are commonly used for analysis of data on costs and length of stay in health facilities [32]. Despite the typically high skewness of cost data, analysis of arithmetic mean is statistically valid, and relevant due to it being proportional to total cost [48]. Other continuous skewed variables, for which gamma GLMs can be used, include serum concentrations of lipids, cytokines or hormones [49,50].

## Conclusions

The method seems most useful for the negative binomial and the gamma distributions which, depending on their parameters, can be highly skewed, making a normal approximation less accurate for the sample mean. Motivated by two biomedical studies, we have shown that the method can be advantageous. Generalized linear models are commonly used to compare means of non-normal distributions and our method is well aligned with this, as well as being simple to use. We hope it will prove useful for situations in which the response variable is expected to be highly skewed, and for which the accuracy of normal approximations are likely to be poor.

## Additional files

Additional file 1:
**Expression of the Berry-Esséen bound in terms of the third non-absolute central moment and a finite sum.**
Additional file 2:
**Third central moments and probability density function for non-Gaussian distributions.**
Additional file 3:
**‘nGLM.r’. R code to implement the methods described in the paper.** It can be opened in any text editor. Instructions are at the top of the file [51,52].

## Abbreviations

CDF: Cumulative distribution functions
GLM: Generalized linear model
HHVI: Human Hookworm Vaccine Initiative
