# Supplementary material for: Sample size calculations for skewed distributions
Source: BMC Med Res Methodol. 2015 Apr 2;15:28. doi: 10.1186/s12874-015-0023-0 (PMC4423589; doi:10.1186/s12874-015-0023-0)
Supplement: Additional file 1: — Expression of the Berry-Esséen bound in terms of the third non-absolute central moment and a finite sum. [file 12874_2015_23_MOESM1_ESM.doc]

**Additional file 1**

To apply the Berry-Esséen theorem, we need to relate a distribution of interest (*Y*) — here assumed discrete — to one with zero mean. Let *Y* be a non-negative discrete random variable with mean $\mu_{Y}$ and variance *σ*^2^>0, and define$R=Y-\mu_{Y}$. Then the third central moment of $R$, denoted by $m_{3}$, can be estimated using *f*, the probability density function of$Y$:

|  | $m_{3}=E\left[ \left( R-\mu_{R} \right)^{3} \right]$ $=E\left[ \left( Y-\mu_{Y}-0 \right)^{3} \right]$ $=\sum_{y\geq0} \left( y-\mu_{Y} \right)^{3}f\left( y \right)$ $=\sum_{y=0}^{\left\lfloor\mu_{Y} \right\rfloor} \left( y-\mu_{Y} \right)^{3}f\left( y \right)+\sum_{y=\left\lfloor\mu_{Y} \right\rfloor+1}^{\infty} \left( y-\mu_{Y} \right)^{3}f\left( y \right)$ |  |
| --- | --- | --- |

with each term in the first sum being negative or zero, and in the second sum being positive. The third *absolute* central moment of *R* can then be expressed as a finite sum as follows:

|  | $m_{3}=E\left[ \left\vert R-\mu_{R} \right\vert^{3} \right]$ $=\sum_{y\geq0} \left\vert y-\mu_{Y} \right\vert^{3}f\left( y \right)$ $=\sum_{y=0}^{\left\lfloor\mu_{Y} \right\rfloor} \left\vert y-\mu_{Y} \right\vert^{3}f\left( y \right)+\sum_{y=\left\lfloor\mu_{Y} \right\rfloor+1}^{\infty} \left\vert y-\mu_{Y} \right\vert^{3}f\left( y \right)$ |  |
| --- | --- | --- |

$$=-\sum_{y=0}^{\left\lfloor\mu_{Y} \right\rfloor} \left( y-\mu_{Y} \right)^{3}f\left( y \right)+\sum_{y=\left\lfloor\mu_{Y} \right\rfloor+1}^{\infty} \left( y-\mu_{Y} \right)^{3}f\left( y \right)$$

$$=E\left[ \left( Y-\mu_{Y} \right)^{3} \right]- 2\sum_{y=0}^{\left\lfloor\mu_{Y} \right\rfloor} \left( y-\mu_{Y} \right)^{3}f\left( y \right)$$

Hence we have obtained the third absolute central moment of *R* in terms of the third (non-absolute) central moment of *Y*, and a finite sum which can readily be calculated numerically. The Berry-Esséen bound in equation (1) for $R=Y-\mu_{Y}$ can now be re-expressed as

$$\frac{C\left\{ E\left[ \left( Y-\mu_{Y} \right)^{3} \right]- 2\sum_{y=0}^{\left\lfloor\mu\right\rfloor} \left( y-\mu_{Y} \right)^{3}f\left( y \right) \right\}}{\left( \sigma^{2} \right)^{\frac{3}{2}}\sqrt{n}}$$
