# Supplementary material for: Sample size calculations for skewed distributions
Source: BMC Med Res Methodol. 2015 Apr 2;15:28. doi: 10.1186/s12874-015-0023-0 (PMC4423589; doi:10.1186/s12874-015-0023-0)
Supplement: Additional file 2: — Third central moments and probability density function for non-Gaussian distributions. [file 12874_2015_23_MOESM2_ESM.doc]

Additional file 2

Third central moments and probability density function for non-Gaussian distributions

| **Distribution** | **Parameters** | **3^rd^ Central Moments [**[**51**](#_ENREF_51)**,** [**52**](#_ENREF_52)**]**$\boldsymbol{E}\left[ \left( \boldsymbol{Y-}\boldsymbol{\mu}_{\boldsymbol{Y}} \right)^{\boldsymbol{3}} \right]$ | **Probability density function,** $\boldsymbol{f}\left( \boldsymbol{y} \right)$ |
| --- | --- | --- | --- |
| Negative  Binomial | $\mu>0$  $k>0$ | $\mu\left( 1+\frac{\mu}{\mu+k} \right)\left( \frac{\mu+k}{k} \right)^{2}$ | $\frac{\Gamma\left( y+k \right)}{\Gamma\left( y+1 \right)\Gamma\left( k \right)}\left( \frac{k}{\mu+k} \right)^{k}\left( \frac{\mu}{\mu+k} \right)^{y}$ |
| Poisson | $\mu>0$ | $\mu$ | $\frac{\mu^{y}}{y!}e^{-\mu}$ |
| Binomial | $0\leq y\leq d$  $0<\mu<1$ | $d\mu\left( 1-\mu\right)\left( 1-2\mu\right)$ | $\left( \begin{matrix} d \\ y \end{matrix} \right)\mu^{y}\left( 1-\mu\right)^{d-y}$ |
| Gamma | $\kappa>0$  $\theta>0$  $\theta\equiv\mu$ | $2\theta^{3}$ | $y^{\kappa-1}\frac{e^{\frac{-y}{\theta}}}{\Gamma\left( \kappa\right)\theta^{\kappa}}$ |
